# Influence of Sunlight and Oral D_3_ Supplementation on Serum 25(OH)D Concentration and Exercise Performance in Elite Soccer Players

**DOI:** 10.3390/nu12051311

**Published:** 2020-05-04

**Authors:** Małgorzata Magdalena Michalczyk, Artur Gołaś, Adam Maszczyk, Piotr Kaczka, Adam Zając

**Affiliations:** 1 Institute of Sport Sciences The Jerzy Kukuczka Academy of Physical Education in Katowice, Poland Mikolowska 72a, 40-065 Katowice, Poland; a.golas@awfkatowice.pl (A.G.); a.maszczyk@awfkatowice.pl (A.M.); a.zajac@awfkatowice.pl (A.Z.); 2 Research and Development Center, Olimp Laboratories Sp. z o.o, 39-200 Dębica, Poland; p.kaczka@olimp-labs.com

**Keywords:** vitamin D, supplementation, soccer, testosterone, speed, power

## Abstract

The aim of this study was to evaluate the influence of natural sun exposure and six weeks of a high dose of vitamin D supplementation on vitamin D, testosterone and cortisol serum concentrations as well as speed, power and VO_2max_ in professional soccer players. Materials: The study was conducted from January to September. At the beginning of the study, 33 professional soccer players were enrolled; however, only 28 subjects (height 181.5 cm; body mass 77.81 ± 8.8 kg; body fat 12.38% ± 2.4% and muscle mass 40.27 ± 5.3 kg) completed the study. The research consisted of three stages. The first one, lasting 10 days, was conducted in January during a training camp in the south part of Cyprus at a latitude of 34 33°, where participants experienced natural sun exposure; it was called a winter sun exposure (WSE) period. The second stage, which was a supplementation period (SP), lasted 6 weeks, during which all subjects were randomly assigned either to an experimental group—EG (*n* = 15)—or a placebo group—PG (*n* = 13)—and were administered 6000 IU/d cholecalciferol or a placebo, respectively. The third stage took place in September, after summertime (summer sun exposure—SSE). The data of the 25(OH)D, free and total testosterone (fT, tT), cortisol as well as 5 and 30 m sprint tests (STs), power of the left leg (PLL) and VO_2max_ were evaluated before and after the WSE period, the SP and SSE. Results: In January, the baseline value of vitamin D in 12 subjects was ≤20 ng/mL, and 14 of them had levels between 20–30 ng/mL and 2 individuals >30 ng/mL. After the WSE period, significant changes in 25(OH)D, fT, tT and cortisol concentration, as well as in the 5 m ST, were observed. After the SP, in the EG, significant changes were found in 25(OH)D, fT, tT and the 5 m ST. Furthermore, a positive correlation between the concentration of 25(OH) fT and tT was observed. After SSE, 2 out of 28 players had <20 ng/mL 25(OH)D, 12 of them had 25(OH)D between 20 and 30 ng/mL and 14 of them had 25(OH)D between 30 and 50 ng/mL. Significant differences in 25(OH)D, fT, tT concentration and the 5 m ST performance were observed following SSE compared with the WSE period. Conclusion: Due to the serum level of 25(OH)D demonstrated by most participants at the beginning of the study and after summertime, all-year-round supplementation with high doses of vitamin D seems to be a reasonable solution to enhance high 25(OH)D concentration in blood and physical performance. In the middle of the winter, almost half of the soccer players were serum deficient of 25(OH)D. After ten days of sun exposure and 6 weeks of vitamin D supplementation, the concentration of 25(OH)D significantly increased, as did testosterone and results in the 5 m sprint test also improved. Therefore, athletes should be constantly monitored for serum levels of 25(OH)D throughout the year and should be supplemented if deficiencies or insufficient amounts of this vitamin occur.

## 1. Introduction

In the past, vitamin D was mainly correlated with the regulation of calcium and phosphate metabolism. In recent years, after the discovery of the receptor for vitamin D (VDR) in most tissues, the interest in the role vitamin D plays in general health has significantly increased [1,2,3,4]. Discovering that vitamin D regulates over 900 genes has completely changed the perception of this compound [5]. It has been proven that vitamin D deficiency significantly affects the functioning of the immune, endocrine and skeletal-muscle systems [6]. The latest guidelines indicate the following serum vitamin D concentration norms: lower than 20 ng/mL as deficiency; between 20 to 30 as insufficiency; between 30 to 50 ng/mL as sufficient or optimal; above 50 to 100 ng/mL as high and above 100 ng/mL as toxic [1,4]. In athletes, a serum 25(OH)D concentration <30 ng/mL may negatively affect aerobic and anaerobic performance, androgen hormone concentration and fat mass [3,7,8]. Frequent injuries and infections in athletes could also be the result of low concentration of 25(OH)D [1,6]. Vitamin D deficiency has proven to be a common symptom in athletes. The primary source of vitamin D, over 90%, is skin synthesis during exposure to solar ultraviolet B (UVB) radiation [6]. Vitamin D can be also delivered via proper nutrition and supplementation [6,9]. In countries located at a latitude >35° N, for several months a year, vitamin D can be supplied only with food and supplements. Unfortunately, vitamin D is found naturally in only a few products, such as fatty fish (e.g., mackerel, salmon, sardines), egg yolks, mushrooms and dairy products, and in relatively small amounts [10].

Vitamin D insufficiency seems to also be a prominent problem among athletes [2,6,7]. It has been estimated that during the winter season, almost 60% of outdoor athletes and 64% of indoor athletes have a level of 25(OH)D <30 ng/mL, which indicates insufficiency of vitamin D [11,12,13,14,15]. Athletes who live at latitudes of 35° degrees or below train outdoors and use sunscreen during summer and cover-up from cold during autumn, winter and spring are vulnerable to serum 25-hydroxyvitamin D (25(OH)D) insufficiency [16,17]. Therefore, optimal sport benefits occur at levels of 25(OH)D above the current definition of sufficiency [12,13]. 

Experimental data has proven that vitamin D plays a crucial role in both muscle strength and post-exercise recovery [1,18]. It reduces the risk of injury caused by inflammation [1,7,18,19], increases cell proliferation and differentiation [5], improves the functioning of the immune system and enhances the anti-inflammatory process [4], and improves muscle strength and power [5]. In the studies of Jastrzebska [7] and Close [14], vitamin D supplementation in a high dose of 5000 IU per day for 8 weeks improved vertical jump height, 10 m sprint times and VO_2max_ in competitive soccer players [7,14]. A high concentration of 25(OH)D in soccer players may determine their aerobic and anaerobic performance capabilities during soccer-specific training such as small-sided games and may improve strength and explosive power [20,21]. These findings imply that athletes who have a vitamin D deficiency after supplementation, which increases its level to the recommended values, could increase their exercise performance and also contribute to faster recovery post-exercise [18]. The effects of 25(OH)D on fat and lean mass in athletes have yet to be determined [11]. Vitamin D concentration is inversely associated with high fat mass [11]. It is supposed that athletes with insufficient 25(OH)D may experience the risk of greater adipose tissue concentration.

VDR and vitamin D-metabolizing enzymes are expressed throughout the entire reproductive male tract, including Leydig cells [22]. Experimental studies have shown that vitamin D deficiency is associated with low testosterone concentrations [3,23]. Lower testosterone concentration has a negative effect on anabolic processes in the athlete’s body. Anabolic testosterone action is stimulated by amino acid uptake and transcriptional regulation of selected genes, which leads to an increase in skeletal muscle protein synthesis [24,25]. Therefore, an elevated level of testosterone combined with resistance training may increase power and speed, which play a significant role in soccer performance. A high testosterone level also enhances post-exercise recovery [25]. Cortisol, as the main catabolic hormone, has an opposite effect. Some authors confirm that oral vitamin D supplementation increases testosterone and at the same time decreases cortisol levels [13,23,26] however, other studies do not confirm such effects [27,28]. 

Most scientists agree that vitamin D supplementation is recommended during the winter season in athletes living in Central and Northern European countries [2,7,8,12]. A significantly lower level of 25(OH)D in players during winter compared with summer has been found [29]. Therefore, the aim of this study was to evaluate the influence of natural sun exposure and a high dose of vitamin D supplementation on hormone concentration, sprinting speed, power and VO_2max_, as well as body mass and body composition in professional soccer players. 

## 2. Material and Methods 

### 2.1. Subjects

The study was conducted from January to September. At the beginning, in January, 33 soccer players from a professional team participated in the study. Five subjects were excluded from the analysis: two were transferred to another club, and three got were injured during the study and did not participate in training and competitions. Finally, 28 players (height 181.5 cm; body mass 77.81 ± 8.8 kg; body fat 12.38% ± 2.4% and muscle mass 40.27 ± 5.3 kg) completed all stages of the research. Exclusion criteria were as follows: vitamin D supplementation one month before the study, vitamin complex supplementation and intestinal disorders that could impair absorption, such as celiac disease, inflammatory bowel disease (IBD) or irritable bowel syndrome (IBS). Subjects were informed about the experimental protocol and procedures and provided written consent to participate in the study. All research procedures were reviewed and approved by the bioethical committee of the Academy of Physical Education in Katowice (ethic references KB-05/2018); furthermore, the study conformed to the tenets of the Declaration of Helsinki for medical research involving human subjects. 

### 2.2. Study Design

**Figure 1:**
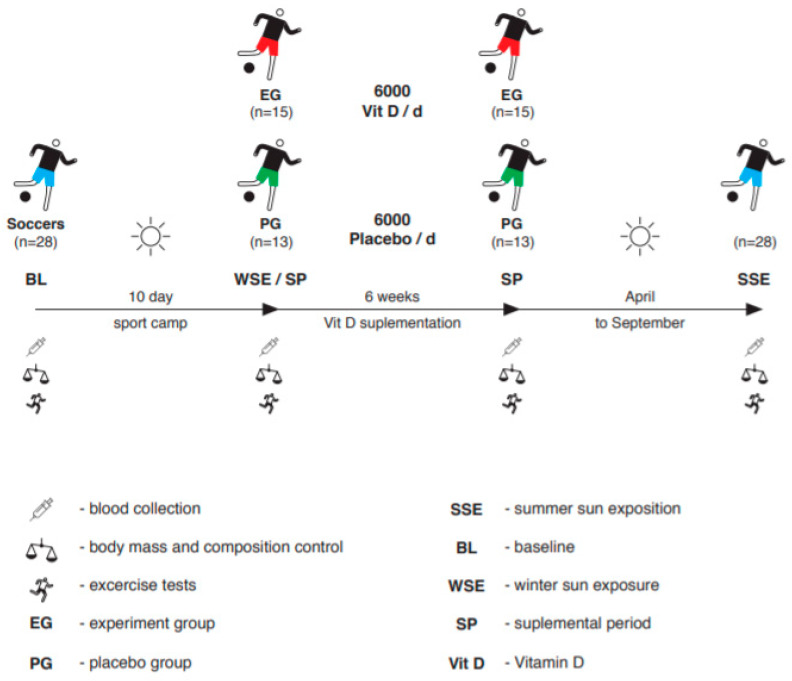
Study protocol.

The study was conducted in three stages (Figure 1). The first stage was carried out in January. The players experienced natural sun exposure (winter sun exposure, WSE) during a 10-day training camp conducted in Cyprus at a latitude of 34 33° N. At that time, the players were exposed to sunlight every day for an average of 8 hours, wearing only T-shirts and shorts. The second stage was conducted immediately after the WSE period and started in late January, lasting 6 weeks until March. At the beginning, subjects were randomly assigned either to an experimental group—EG (*n* = 15)—or a placebo group—PG (*n* = 13). The EG was supplemented with 6000 IU/d cholecalciferol, while the PG was administered a placebo. The third stage of the experiment took place in September, after summertime (summer sun exposure—SSE). It should be mentioned that the first and the second stages of the study were performed in the middle of wintertime in Poland at an altitude of 50° N. During this period, two main factors limit skin contact with sunlight and finally inhibit vitamin D secretion. The first is low temperature, which makes soccer players train fully clothed so that their body surface is completely covered, and the second is that at this time of the year, from January to March, most days are overcast, which significantly limits sun exposure. In turn, on sunny days the angle of incidence is different, which is also not conducive to skin 25(OH)D synthesis. The players were indicated not to use sunscreen protection during the study. At every stage of the study, biochemical variables, i.e., 25(OH)D, testosterone and cortisol, as well as body mass and body composition, were evaluated. Additionally, all participants performed the sprint, power of the left leg (PLL) and VO_2max_ tests. Before the tests, a 5-minute warm-up was performed. All tests were performed in an accredited laboratory.

### 2.3. Supplementation

Each day for 6 weeks, after supper, the players from the EG consumed 3 capsules containing 2000 IU cholecalciferol (Gold Vit D_3_, Olimp Labs, Poland), while the players from the PG consumed 3 capsules with 2000 UI of sunflower oil. All capsules looked identical. Vitamin D (Gold Vit D_3_) and placebo capsules were prepared, donated and provided with a quality control analysis by Olimp Laboratories Sp. z o.o (Poland). During the study, the subjects were asked to maintain their regular diet. The diet of the players was a standard mixed diet which they maintained throughout the entire season.

### 2.4. Training Program

During the training camp in Cyprus, the soccer players had seven training sessions per week on the field with additional resistance training sessions twice a week indoors. The resistance training sessions included the following three exercises: the bench press, back squats and pull-ups. The athletes performed 10 sets of 10 repetitions of each exercise. During this time, three scrimmage matches were played. 

During the SP, the soccer players trained six times a week on the field. Twice a week, they performed resistance training, using the same exercises as during the training camp, although they performed five sets of five repetitions in order to increase explosive strength and power. Once a week, they played a scrimmage match. 

### 2.5. Sprint Test

The test was conducted on the field using Microgate photo cells (Photocells Witty Gate, Bolzano, Italy) located at the 5th and 30th m. Both sprint tests were conducted on the same day and were performed twice, with a 5-minute rest interval between trials. 

### 2.6. PLL Test 

The test was performed on the Keiser Leg Press machine (Model 2531, Keiser Corporation, Fresno CA, USA) and comprised two stages. The first one consisted of 5 to 7 attempts with a progressive load. The second stage involved performing a maximum extension of the lower limbs twice in order to determine the maximum explosive strength with a load of 50% 1 RM.

### 2.7. VO_2max_ Test

The athletes ran on a treadmill (HP Cosmo, COS 10170, Traunstein, Germany) with the starting speed of 6 km/h with speed increases of 2 km every 3 minutes until volitional exhaustion [30]. Oxygen uptake was monitored throughout the test. 

### 2.8. Biochemical Analysis

Fasting blood samples were collected in the morning (around 8:00 am), after body mass analysis. Vacutainer tubes were used to determine the vitamin D and hormone profile. Blood serum was separated using routine procedures and either processed immediately or kept frozen at −70 °C until further analysis. Serum 25(OH)D and hormone concentrations were assessed by radioimmunoassay diagnostic kits. 25OH-Vitamin D was determined by RIA-CT KIP1971/KIP1974 (DIAsource ImmunoAssays SA, Louvain-la-Neuve, Belgium). Total testosterone was evaluated using the [I-125] RIA kit (Ref: RK-61CT) (Institute of Isotopes Ltd, Budapest, Hungary), free testosterone was assessed by Active Free Testosterone RIA DSL4900 (Beckman Coulter, Barueri, Brasil) and cortisol using Cortisol - RIA-CT KIPI28000 (DIAsource ImmunoAssays SA, Louvain-la-Neuve, Belgium). 

### 2.9. Body Mass and Body Composition Evaluation 

After an overnight fast and a minimum of 3 days without alcohol consumption, each subject reported to the laboratory in the morning for blood analyses and body mass evaluation, as well as aerobic and anaerobic tests. The evaluation of body composition and adipose tissue content was performed by electrical impedance analysis (MF-BIA) using the InBody 720 (Biospace Co., Ltd., Seoul, Korea). The measurements were taken under laboratory conditions, according to the instructions of the manufacturer. 

### 2.10. Statistics Analysis

The Shapiro–Wilk test was used to verify the normality of the sample’s data variances. ANOVA with repeated measures was used to evaluate differences between the analyzed values before and after the sun exposure as well as before and after D_3_ supplementation as between baseline (BL) and SSE. When appropriate, a Tukey’s post hoc test was used to compare selected data. The effect size (eta-squared; η^2^) of each test was calculated to determine the significance of results. The effect size was classified according to Hopkins as 0.01—small, 0.06—medium, 0.14—large [31]). Differences between stages were calculated using one-way ANOVA and Tukey’s post hoc tests. To evaluate the correlation between the study variables and vitamin D level the Pearson correlation coefficient was used. Statistical significance was set at *p* < 0.05. All statistical analyses were performed using Statistica 15 (Stat Soft, Inc., Kraków, Poland, 2018) and Microsoft Office (Version 2015, Warsaw, Poland Group), and results were presented as means with standard deviations.

## 3. Results 

**Figure 2:**
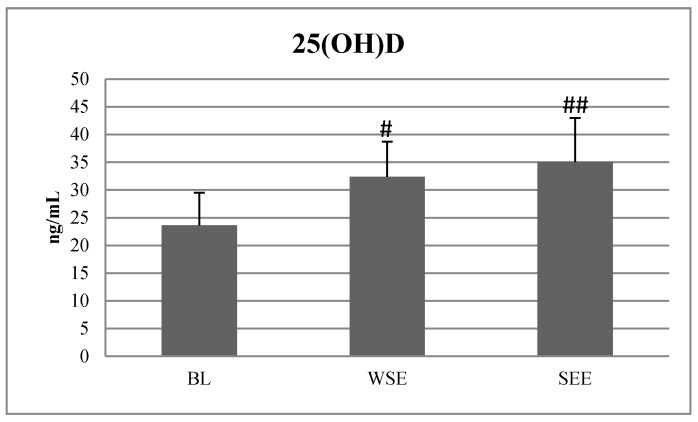
25(OH)D serum concentration in different periods of the scoccer season. Note: BL—baseline, WSE—after winter sun exposure, SEE—after summer sun exposure. #—significant difference compared to BL (*p* < 0.05), ##—significant difference compared to BL (*p* < 0.01).

**Figure 3:**
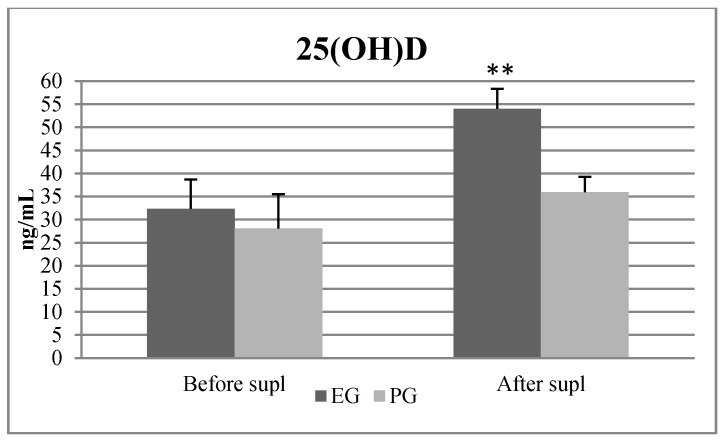
25(OH) D serum concentration before and after supplementation. Note: EG—experimental group, PG—placebo group. **—significant difference compared to before supplementation (*p* < 0.01).

Out of thirty-three subjects who initially entered the study, only twenty-eight completed the entire experiment. Figure 2 and Figure 3 and Table 1, Table 2 and Table 3 present the results. At BL, none of the 28 soccer players exceeded the 25(OH)D content up to 40 ng/mL, 12 subjects had vitamin D level ≤20 ng/mL, 14 of them were between 20 to 30 ng/mL and 2 of them reached vitamin D level >30 ng/mL. After the SP, in the EG, 12 out of 15 players exceeded 50 ng/mL of 25(OH)D. After SSE, none of the 28 participants reached the level above >50 ng/mL of the 25(OH)D. Two out of 28 players had <20 ng/mL 25(OH)D, 12 of them had 25(OH)D between 20 and 30 ng/mL and 14 of them had 25(OH)D between 30 and 50 ng/mL. 

The ANOVA with repeated measures revealed statistically significant differences between BL, WSE and SSE in 25(OH)D fT, tT and cortisol concentration. After the WSE period, there were significant differences and large size effects observed between 25(OH)D, fT, tT cortisol and 5 m ST values, compared with BL values. The post hoc tests revealed significantly higher values of 25(OH)D (F = 15,920, *p* = 0.003; η^2^ = 0.423), fT (F = 35,241, *p* = 0.001; η^2^ = 0.335), tT (F = 8371, *p* = 0.007; η^2^ = 0.154), cortisol (F = 5.138, *p* = 0.031; η^2^ = 0.231), and a lower value of the 5m sprint test (F = 4.37, *p* = 0.044; η^2^ = 0.056), (Figure 2, Table 1 and Table 2). There were no significant differences in 25(OH)D between the EG and the PG before supplementation (Figure 3). After the SP, in the EG, the repeated measures ANOVA revealed statistically significant differences between before and after vitamin D supplementation in 25(OH)D, fT, tT concentration and in the 5m ST. Post hoc tests revealed statistically significant increases and large effect sizes in 25(OH)D values (F = 17,003, *p* = 0.001; η^2^ = 0.532), fT (F = 12,889, *p* = 0.001; η^2^ = 0.2893), and tT (F = 7626, *p* = 0.015; η^2^ = 0.212) as well as the 5m ST (F = 4.35, *p* = 0.047; η^2^ = 0.055). Additionally, the Pearson coefficients confirmed a positive correlation between 25(OH)D and fT and tT (Table 4). ANOVA revealed statistically significant differences in vitamin D concentration after SSE. The post hoc tests indicated a statistically significant increase and large effect sizes in SSE values of 25(OH)D (F = 15,543, *p* = 0.001; η^2^ = 0.533), fT (F = 33,126, *p* = 0.001; η^2^ = 0.298) and cortisol (F = 33,085, *p* = 0.001; η^2^ = 0.351) after SSE compared to BL and WSE values. The ANOVA with repeated measures revealed no statistically significant differences after stage I, II and III in body fat and muscle mass were observed (Table 3).

The one-way ANOVA revealed significant differences between particular stages of serum 25-hydroxyvitamin D levels upon supplementation or sun exposure, dividing the players into deficient, insufficient or sufficient level. The post hoc tests indicated significant differences between BL and WSE for all groups with *p* = 0.002 and in SG between before and after for all groups with *p* = 0.001.

## 4. Discussion

The main purpose of this study was to assess how sun exposure or supplementation of vitamin D affects the levels of 25(OH)D, testosterone and cortisol concentration, body mass and body composition, sprint speed at 5 and 30 m, power of the left leg and VO_2max_ in elite soccer players. The study consisted of three stages. During the first stage, the impact of natural sun exposure during a 10-day training camp at the latitude of about 35° was evaluated. During the second stage, we measured the impact of a high dose, i.e., 6000 IU/day, of cholecalciferol administered for 6 weeks, and finally, during the third stage, the impact of summer sun exposure was considered. It was confirmed that 10 days of sun exposure and 6 weeks of vitamin D supplementation significantly increased 25(OH)D blood concentration (Figure 2). 

During the first stage of the research, our participants presented an insufficient serum level of vitamin D, which was below 30 ng/mL. This result shows that during winter, soccer players had low skin synthesis and insufficient dietary supply of vitamin D [6,7,8]. Such a low level may cause deficits in strength, power and endurance but also may result in degeneration of type II muscle fibers and a slower rate of post-exercise recovery [6,32]. In January, after a 10-day training camp in Cyprus, where soccer players were exposed to the sun for 8 hours daily, the 25(OH)D significantly increased from 23.63 ± 5.9 ng/mL to 32.40 ± 6.3 ng/mL, which met the lower limit of sufficiency of this vitamin [12,13]. Moreover, after 6 weeks of cholecalciferol supplementation, blood vitamin D increased from 32.37 ± 6.3 ng/mL to 54.03 ± 7.4 ng/mL, which indicated reaching the lower limit for high concentration of vitamin D. Finally, after the summer season, 25(OH)D serum concentration was 35.08 ± 7.9 ng/mL, which was significantly higher than in January. However, we noticed that, after SSE, despite having spent several months training outdoors, often in the sun, most players demonstrated not high but a normal range of (30–50 ng/mL) of vitamin D serum concentration [12]. Our results show that after SSE, two out of 28 subjects had a deficit <20 ng/mL of 25(OH)D. These results, combined with the ones from January (Figure 2 and Figure 3), confirmed that soccer players should be supplemented with high doses of vitamin D throughout the year. Similar results were presented by Halliday et al [33]. They reported that 25(OH)D concentrations changed across time and averaged 49.0 ± 16.6, 30.5 ± 9.4 and 41.9 ± 14.6 ng/mL in the fall, winter and spring, respectively, in 12 indoor and 29 outdoor collegiate athletes. In turn, after the 6-week SP, significant increases in 25(OH)D in the EG were observed compared with the PG. These results are in accordance with those presented by other authors [7,28,34]. Jastrzebska et al. [7] showed that after 8 weeks of 5000 IU/d oral vitamin D supplementation in soccer players, the level of 25(OH)D increased significantly from 48.5 ± 8.6 ng/mL to 106.3 ± 26.3 ng/mL [7]. Texeira et al. [35] also recorded an increase in 25(OH)D from 10.6 ng/mL to 43.4 ng/mL after 8 weeks of supplementation, but not above 50 ng/mL, which still indicated an insufficient amount [12]. Compared with another study, Texeira et al. [35] used a lower dose of cholecalciferol, 25,000 IU per week, which amounted to about 3666 IU/d. In our study, the subjects reached a value of 54.03 ± 7.4 ng/mL, which is slightly above the sufficient limit of reference values, which is 30 to 50 ng/mL. 

We expected a higher increase of vitamin D, but the supplementation time was shorter than in other studies [7,28,34]. We had limited time to conduct our study, which resulted from the fact that our research group included elite soccer players. We had to finish the research in March since our participants started playing regular league matches. The coaching staff did not agree to an extension of the research. They argued that many players were stressed by frequent measurements and blood sampling. In addition, exercise tests significantly disrupted training concepts. A longer period of supplementation of another 2 weeks would probably allow us to achieve higher values of 25(OH)D, like in other studies [7,20]. Additionally, other factors, which were not considered in the current study, could have had an impact on the absorption of vitamin D from the digestive tract into the bloodstream. The microbiota composition or inflammation of the intestines could individually decrease cholecalciferol absorption [36,37]. Our data confirm previous results, where oral vitamin D supplementation has been suggested as an alternative to obtain adequate levels of 25(OH)D_3_ all year round, but especially during the winter season [2,7,20]. 

25(OH)D converted to its biological active form, 1.25-dihydroxyvitamin D (1.25(OH)2D) binds to their receptor -VDR, located in most tissues, and is translocated into the cell [1,5]. The complex hormone receptor interacts with retinoid X receptor (RXR) and in the form 1.25(OH)2D-VDR –RXR is translocated into the nucleus where it binds to vitamin D response elements (VDREs) and modulates the expression of numerous genes [1,5]. Active complex 1,25(OH)_2_D and VDR increase expression of IGF and IGF-BP_3_ genes and inhibit interleukin IL-12, TNF-α or IF-γ genes’ expression [1,5,19]. The vitamin D receptor and the vitamin D metabolizing enzymes are expressed throughout the male reproductive system, indicating their modulation of reproductive function [6,27]. In this study we observed that after the WSE period and after SSE, when 25(OH)D significantly increased, and free and total testosterone concentration also increased (Table 2). Previous data confirm our results [26,38,39]. Lombardi found [38] that higher 25(OH)D levels were associated with higher testosterone and lower cortisol levels in 45 soccer players. In addition, Pilz et al. [26] observed higher levels of testosterone in men who had been supplemented with 3332 IU/day of vitamin D for one year. Nimptchs et al. [22] explained the increase in testosterone levels after supplementation with vitamin D with the fact that both the receptors for vitamin D and the enzymes involved in its metabolism can be detected in human Leydig cells, which produce testosterone. This suggests that vitamin D may locally affect the production of this hormone. Interestingly, there was a relationship between vitamin D levels and the amount of sperm and their motility. Additionally, this hypothesis was supported by the results of studies in genetically modified mice, which have no receptor for vitamin D and have suffered gonadal failure leading to hyper-gonadotrophic hypogonadism as well as reduced sperm count and motility. The study authors conclude that their results support the results of several previous works, which exhibit a positive correlation between levels of vitamin D and testosterone levels [39,40]. Our results are interesting because we observed both vitamin D and testosterone increases. Both of them are associated with muscle mass, strength and power [6,25]. Higher testosterone secretion increases intramuscular protein synthesis, which was observed after vitamin D supplementation (muscle mass: 40.25 ± 3.4 vs. 44.15 ± 4.5 kg) in the EG, and indirectly influences 5 m ST results (1.09 ± 0.04 vs. 1.04 ± 0.05) [6,25]. The capability of testosterone to increase the muscle cross-sectional area, both solely and in combination with resistance exercise has been confirmed [24]. Athletes with higher testosterone concentration and adequate protein consumption can increase muscle synthesis, increase muscle fibre recruitment and finally increase total training loads [24,25]. 

1.25(OH)2D, as a biological active form of 25(OH)D, is involved in muscular development and function, control of hormonal systems and control of inflammation and tissue and muscle damage [5]. The main catabolic hormone causing muscle damage in athletes is cortisol [25]. Therefore, an increase in serum 25(OH)D levels could decrease cortisol secretion and stress levels [19]. Regarding this variable, after the WSE period, despite a significant 25(OH)D increase, there was also a significant cortisol increase. A high cortisol concentration in athletes is a common state and indicates stress, exercise overloading and inadequate muscle recovery [1,41]. In our study, an increased cortisol level (Table 1) probably resulted from the fact that the study subjects trained intensively and played matches throughout the experiment. Such conditions are undoubtedly conducive to the occurrence of stress, both physical and mental, resulting from training and competition [42,43]. Under such conditions, there is a significant secretion of cortisol in the adrenal glands, which undoubtedly increases catabolism and inhibits the regeneration process [42].

It is widely known that supplementation with high doses of 5000–10,000 IU/day of vitamin D_3_ is effective in treating athletes with 25(OH)D deficiency [9,14,42]. Soccer is characterized by the continuous combination of short sprints, rapid accelerations/decelerations and changes of direction interspersed with jumping, kicking, tackling and informal times for recovery [44,45]. Distances of approximately 10 km covered during the match at different intensities are able to stimulate both the aerobic and anaerobic systems [44,45]. By considering the duration of the game (70–90 min) and the values of maximum oxygen uptake, soccer game appears to be dependent upon the aerobic system [45]. In this sense, a high correlation was found between aerobic capacity and high vitamin D concentration, both naturally and as a result of supplementation [7]. Therefore, the vitamin D level is associated with endurance performance [9]. The effects of a high dose of vitamin D_3_ supplementation (5000 IU/day) for eight weeks on aerobic capacity expressed as VO_2_max was demonstrated by Jastrzebska et al. [7] in professional Polish soccer players who showed a significant increase in VO_2max_. In previous research, it has been proven that supplementing soccer players with vitamin D improves their aerobic capacity [7] as well as speed and explosive power [2]. In Jastrzebska’s [7] study, after a dose of 5000 IU/day and after high-intensity soccer training, subjects improved their VO_2max_ values by 20%. Koundourakis et al. [44] also found a significant linear relationship between VO_2max_ and the level of vitamin D in professional soccer players. For that reason, vitamin D supplementation could influence VO_2max_ values given that CYP enzymes that activate vitamin D_3_ have proteins with heme groups, which could potentially increase the affinity of oxygen binding to hemoglobin [5]. Conversely Książek et al. [32] did not find any significant relationship between muscle strength, VO_2_max and highest vitamin D deficiency in professional soccer players from Poland tested during winter. In our study, just like in the Książek et al.’s research, we did not find any significant changes in VO_2_max at any stages of the study. Although we noticed that after 10 day of sun exposure, VO_2_max increased slightly from 56.57 ± 3.9 to 58.00 ± 4.9 and after the SP in the EG from 57.8 ± 4.8 to 58.92 ± 5.3 ml/kg/min (Table 2).

Vitamin D signals the expression of the size of myotubes to make them larger, and as a result, muscle strength increases [32,43]. It was documented that vitamin D supplementation increased recovery of muscle after injury or damage [45]. In subjects with initially low serum of 25(OH)D, there was an increase in pro-inflammatory e.g., TNF-α and IL-6 cytokine, secretion [19,45]. In athletes, those cytokines intensify damage in muscle and enhance the risk of injury [19,45]. In this study, after the WSE period and vitamin D supplementation, some changes in speed were observed, but none in explosive strength. After the WSE period, we noticed significant changes in 5 m sprint speed results: from 1.13 ± 0.05 s to 1.09 ± 0.04 s and also after 6 weeks of 6000 IU/d of cholecalciferol supplementation from 1.09 ± 0.04 s to 1.04 ± 0.05 s. The observed increase in muscle mass (Table 3) could indirectly affect the speed improvement. Vitamin D induces myogenesis and muscle protein synthesis causing increases in fast-twitch muscle cells [1,6,26]. This type of fibres are the major determinant of explosive strength, which results in high power output and fast muscle contractions [32,44]. 

## 5. Conclusions 

According to the findings of the current study, although the study participants were professional soccer players, in most of them, the level of vitamin D was insufficient in the middle of the winter season, but also after the summertime. Vitamin D deficiency adversely affects the muscle, immune and hormonal systems. After 10 days of sun exposure and after 6 weeks of vitamin D supplementation, when 25(OH)D blood concentration increased, the soccer players achieved better results at the 5 m speed test and higher testosterone concentrations. This information may be valuable for sport scientists, coaches and players to enhance soccer performance and consider vitamin D supplementation. The obtained results confirm that vitamin D is very important for the muscle and hormonal system. Therefore, athletes should be constantly monitored for 25(OH)D blood levels throughout the year and should be supplemented if deficiencies or insufficient amounts occur.

## 6. Strengths and limitations

We are aware that our study has strong and weak points. The strong point was the possibility to study a group of professional soccer players, access to whom is severely limited. We also had the opportunity to conduct a longitudinal study, which lasted from January to September, and we collected biochemical and physical fitness variables four times during the season. Unfortunately, the limitations include a small number of subjects, which is the result of a specific sport discipline. In soccer, during the season, team composition changes due to transfers and injuries. In addition, it was possible to diagnose inflammatory bowel markers during the supplementation period. The results indicate that if there were additional negative factors they could have affected the absorption of vitamin D.

## Figures and Tables

**Table 1:** Changes in hormones serum concentration in different stages of the study.

Variables	BL	WSE	EG		PG		SSE
			Before supl	After supl	Before supl	After supl	
fT, pg/mL	10.16 ± 3.2	18.78 ± 5.3#	18.66 ± 5.3	28.86 ± 3.6*	18.21 ± 4.9	23.20 ± 5.2	20.84 ± 4.9##
tT, nmol/L	19.22 ± 4.8	23.86 ± 6.5#	22.95 ± 6.4	28.25 ± 3.2*	23.64 ± 3.9	26.60 ± 5.1	20.42 ± 5.3
Cortisol, μg/L	357.61 ± 133.0	469.47# ± 142.4	469.50 ± 142.4	517.38 ± 127.8	512.44 ± 103.6	572.750 ± 227.12	739.53## ± 180.1

Note: fT—free testosterone, tT—total testosterone, BL—baseline, WSE—winter sun exposure, SSE—summer sun exposure, EG—experimental group; PG—placebo group, supl—supplementation *—*p* < 0.05- significant difference compared with the before supplementation, #—*p* < 0.05—significant difference compared with the BL, ##—*p* < 0.01—significant difference compared with the BL.

**Table 2:** Results of aerobic and anaerobic performance in different stages of the study.

Variables	BL	WSE	EG		PG		SSE
			Before supl	After supl	Before supl	After supl	
VO_2max_, ml/kgO_2_/min	56.57 ± 3.9	58.00 ± 4.9	57.8 ± 4.8	58.92 ± 5.3	58.0 ± 3.4	59.0 ± 3.9	58.05 ± 4.6
5m ST, s	1.13 ± 0.05	1.09 ± 0.04#	1.09 ± 0.04	1.04 ± 0.03#	1.08 ± 0.06	1.06 ± 0.07	1.11 ± 0.06
30m ST, s	4.20 ± 0.08	4.15 ± 0.07	4.16 ± 0.08	4.09 ± 0.09	4.16 ± 0.09	4.09 ± 0.1	4.16 ± 0.07
PLL, watt	1600.76 ± 124.7	1665.83 ± 116.7	1665.84 ± 116.7	1720.61 ± 113.2	1610.25 ± 11.8	1685.00 ± 96.2	1672.90 ± 112.8

Note: ST—speed test, PLL—power of the left leg, BL—baseline, WSE—after winter sun exposure, SEE—after summer sun exposure, EG—experimental group, PG—placebo group, #—*p* < 0.05—significant difference compared with the BL.

**Table 3:** Changes of body mass and body composition during the study.

Variables	BL	WSE	EG		PG		SSE
			Before supl	After supl	Before supl	After supl	
Body mass, kg	77.81 ± 8.8	78.74 ± 8.4	78.74 ± 9.4	80.08 ± 8.5	75.47 ± 7.0	77.37 ± 6.0	80.60 ± 9.9
Body fat, %	12.38 ± 2.4	10.77 ± 1.4	10.67 ± 2.2	7.69 ± 2.4	9.87 ± 2.8	7.49 ± 2.8	7.5 ± 2.6
Muscle mass, kg	40.27 ± 5.3	41.52 ± 4.2	40.25 ± 3.4	44.15 ± 4.5	40.62 ± 5.5	42.16 ± 5.9	40.70 ± 5.2

Note: BL—baseline, WSE—after winter sun exposure, SSE—summer sun exposure, EG—experimental group, PG—placebo group.

**Table 4:** Pearson correlation coefficients in the EG after supplementation.

Variables	Pearson correlation
	25(OH)D
Body fat	0.341
Muscle mass	0.397
VO_2max_	0.017
5 m ST	−0.054
30 m ST	−0.052
PLL	0.021
Cortisol	−0.237
fT	0.638
Tt	0.609
